# Activation of ERK and NF-κB during HARE-Mediated Heparin Uptake Require Only One of the Four Endocytic Motifs

**DOI:** 10.1371/journal.pone.0154124

**Published:** 2016-04-21

**Authors:** Madhu S. Pandey, Colton M. Miller, Edward N. Harris, Paul H. Weigel

**Affiliations:** 1 Department of Biochemistry & Molecular Biology, University of Oklahoma Health Sciences Center, Oklahoma City, OK 73104, United States of America; 2 Department of Biochemistry, University of Nebraska, Lincoln, NE 68588, United States of America; Institut Curie, FRANCE

## Abstract

Fifteen different ligands, including heparin (Hep), are cleared from lymph and blood by the Hyaluronan (HA) Receptor for Endocytosis (HARE; derived from Stabilin-2 by proteolysis), which contains four endocytic motifs (M1-M4). Endocytosis of HARE•Hep complexes is targeted to coated pits by M1, M2, and M3 (Pandey *et al*, Int. J. Cell Biol. 2015, article ID 524707), which activates ERK1/2 and NF-κB (Pandey *et al* J. Biol. Chem. 288, 14068–79, 2013). Here, we used a NF-κB promoter-driven luciferase gene assay and cell lines expressing different HARE cytoplasmic domain mutants to identify motifs needed for Hep-mediated signaling. Deletion of M1, M2 or M4 singly had no effect on Hep-mediated ERK1/2 activation, whereas signaling (but not uptake) was eliminated in HARE(ΔM3) cells lacking NPLY^2519^. ERK1/2 signaling in cells expressing WT HARE(Y2519A) or HARE(Y2519A) lacking M1, M2 and M4 (containing M3-only) was decreased by 75% or eliminated, respectively. Deletion of M3 (but not M1, M2 or M4) also inhibited the formation of HARE•Hep•ERK1/2 complexes by 67%. NF-κB activation by HARE-mediated uptake of Hep, HA, dermatan sulfate or acetylated LDL was unaffected in single-motif deletion mutants lacking M1, M2 or M4. In contrast, cells expressing HARE(ΔM3) showed loss of HARE-mediated NF-κB activation during uptake of each of these four ligands. NF-κB activation by the four signaling ligands was also eliminated in HARE(Y2519A) or HARE(M3-only;Y2519A) cells. We conclude that the HARE NPLY^2519^ motif is necessary for both ERK1/2 and NF-κB signaling and that Tyr^2519^ is critical for these functions.

## Introduction

Heparin (Hep) is a highly sulfated anionic glycosaminoglycan consisting of repeating disaccharide units, containing N-acetylglucosamine and glucuronic acid or iduronic acid. Hep binds to many different matrix components, cell surface and soluble proteins; *e*.*g*. 22% of total plasma proteins bind to Hep [1]. Hep also binds to many growth factors (*e*.*g*. FGF-2, PDGF and HGF) and their receptors [2,3]. Unfractionated high mass Hep (UFH) and low mass Hep (LMWH) are highly prescribed anticoagulant drugs for prevention and treatment of thromboembolic diseases [4]. Hep has also been clinically used to prevent recurrent early pregnancy loss due to antiphospholipid syndrome, a complication associated with blood clotting in pregnant mothers that leads to miscarriage [5].

The activities of HARE and Stab2 (the full-length receptor) were discovered in the early 1980s by Laurent and co-workers [6–10] as the clearance receptors that remove HA from the vascular and lymphatic circulatory systems. HARE and Stab2 are highly expressed in the sinusoidal endothelial cells (SECs) of liver and lymph node [11–14], the main organs responsible for systemic clearance of multiple ligands from blood and lymph fluid, respectively. HARE and Stab2 function as the primary scavenger receptors for the systemic clearance of HA, Hep [15,16] and 13 other ligands [13,17–24] including phosphatidylserine (apoptotic cells), dermatan sulphate (DS), chondroitin, chondroitin sulfate types A, C, D and E, and oxidized or acetylated LDL (AcLDL). The 190-kDa HARE isoform is generated by proteolysis of the full-length Stab2 receptor and is not a splice variant [25,26]. HARE begins at Ser^1135^ and ends at the C-terminal Leu^2551^ of full-length Stab2 [13,27]; therefore, both proteins are functional endocytic receptors with different N-terminal domains and identical C-terminal, transmembrane and cytoplasmic domains. HARE is expressed more highly than full-length Stab2 in liver and spleen, with about two-thirds of Stab2 processed to HARE [12,14].

Macrophages express HARE/Stab2, which serve as apoptotic cell receptors [28]; these mobile macrophages in tissues and immobile SECs in lymph node and liver provide complementary multi-ligand clearance systems. The major function of these constitutively recycling scavenger receptors is to remove their ligands by coated pit-mediated endocytosis and deliver them to SEC lysosomes or macrophage phagosomes for degradation [29–31]. The two receptors are highly expressed in spleen [11] and bone marrow [32], where their function is less obvious; perhaps mediating local ligand turnover in tissues undergoing matrix remodeling. HARE/Stab2 are also expressed in corneal and lens epithelium, mesenchymal heart valve cells, ependymal brain ventricle cells, prismatic epithelial cells covering renal papillae, and oviduct [33]. In lymph nodes, and likely in liver and bone marrow as well, HARE is a homing receptor for metastatic tumor cells with surface HA coats [34], which is a common phenotype of aggressive cancers.

It has become clear that HARE and Stab2 have additional functions other than just ligand clearance. These receptors also respond to a sub-set of ligands by stimulating cell signaling pathways leading to activation of ERK1/2 and NF-κB and downstream gene expression changes [35,36]. Park *et al*. [28] found that phagocytosis of apoptotic cells by activated macrophages is mediated by HARE/Stab2 binding to phosphatidylserine and this interaction stimulates signaling that leads to the synthesis and release of TGF-β, an anti-inflammatory cytokine. HA, Hep, DS and AcLDL show very similar profiles for ERK1/2 and NF-κB activation, but chondroitin sulfate types A, C, D and E are not signaling competent [37]. Hep activation of NF-κB mediated gene expression occurs with an apparent K_m_ of 20 nM for the cellular response, consistent with the binding affinity of Hep for HARE (K_d_ ~20–60 nM). Small-to-intermediate mass HA can induce cell signaling pathways and modulate biological responses such as angiogenesis, wound healing and tumorigenesis [38–40]. Although HARE binds and internalizes all sizes of HA, only binding to 40–400 kDa HA (not smaller or larger sizes of HA) stimulates HARE-mediated cell signaling, leading to activation of ERK1/2 and NF-κB-mediated gene expression [35,36]. These findings support a role of HARE•ligand signaling as part of a proposed “Tissue-Stress Sensor” system during various physiological challenges [29].

The HARE cytoplasmic domain (CD) contains four endocytic motifs (M1, M2, M3 and M4) that target HARE•ligand complexes to coated pits; M1, M3 and M4 mediate HA uptake [41], whereas M1, M2 and M3 mediate Hep endocytosis [42]. Here we used stable cell lines expressing a panel of CD mutants to investigate which of the three motifs involved in HARE•Hep complex internalization is needed to activate cell signaling leading to increased ERK1/2 phosphorylation and NF-κB activation. We found that HARE and ERK1/2 are in complexes during Hep uptake and that only the M3 motif (NPLY^2519^) was essential for ERK1/2 activation. Deletion of the HARE M3 motif or mutation of Tyr^2519^ also decreased or eliminated NF-κB activation leading to gene expression stimulated by Hep as well as HA, DS and AcLDL.

## Materials and Methods

### Cells, plasmids and reagents

Stable Flp-In 293 (HEK) cell lines expressing 190 kDa wildtype (WT) HARE, various CD mutants of HARE or empty vector (EV) were created as previously described [16,35,41]. Flp-In 293 cells do not express endogenous HARE mRNA or protein and they have a single unique recombinase-mediated gene insertion site [43,44]. Thus, all the cell lines used had only a single insertion at this same correct site [45] and the cohort of cell lines used is as genetically identical as can be obtained. Lipofectamine 2000, Lipofectamine LTX and PLUS reagents, glutamate, zeocin, hygromycin B, DMEM and Fetal Bovine Serum (FBS) were from Invitrogen (Carlsbad, CA). Dual Luciferase (LUC) Reporter Assay System (E1960; Plasmid vector pGL4.32[luc2P/NF-κB-RE/Hygro]) and Luminometer Glomax 20/20 were from Promega (Madison, WI). *Renilla* luciferase Plasmid pRL-TK was a gift from Dr. K. Mark Coggeshall (Oklahoma Medical Research Foundation). UFH (unfractionated) was from Celsus (Cincinnati, OH) or Sigma (St. Louis, MO) and LMWH (Lovenox) was from Baxter Pharmaceuticals, LLC (Bloomington, IN). Based on size-exclusion chromatography coupled to multi-angle laser light scattering [15], the weight-average molar masses of the preparations used were 13.5 kDa (UFH) and 3.5 kDa (LMWH). Goat anti-V5 polyclonal antibody (Ab; IgG) was from Bethyl Labs (Montgomery, TX). Rabbit anti-phospho-ERK1/2 (p44/42; Thr-P(202) and Tyr-P(204), anti-ERK1/2 and mouse anti-actin Abs were from Cell Signaling (Beverly, MA). Goat anti-rabbit IgG-HRP, donkey anti-goat IgG-HRP, and donkey anti-mouse IgG-HRP were from Santa Cruz Biotechnology (Dallas, TX). Other materials, reagents, and kits were obtained as described [35]. Unless specified, all other reagents were the highest purity grade available from Sigma (St. Louis, MO). Complete Medium contained DMEM plus 8% FBS and 100 μg/ml hygromycin B. Preincubation Medium was DMEM without FBS or hygromycin. Lysis Buffer contained 20 mM Tris, pH 7.2, 1 mM sodium orthovanadate, 3 mM benzamidine, 2 mM sodium pyrophosphate, 5 mM sodium fluoride, 2 mM EGTA, 5 mM EDTA, 1 μg/ml of protease inhibitor cocktail (#P8340; Sigma, St. Louis MO) and 0.5% (v/v) Nonidet P-40. Transfection medium, blocking buffer, PBS, stripping buffer, and other buffers were described previously [36,37].

### Cell Culture and Hep stimulation of ERK1/2 Activation

Cells were grown in complete medium till confluence and then plated in individual 35 mm tissue culture plates as described [46] and grown for at least 2 days (to 80–90% confluence) before experiments. Cells were washed with sterile PBS, incubated in fresh medium without serum for 1 h at 37°C, washed and then incubated at 37°C in fresh serum-free medium containing 10 μg/ml (709 nM) UFH for the indicated times. Time-zero values were in the absence of heparin or other ligands. Cells were then washed with ice-cold PBS and lysed with Lysis Buffer. The cell lysates were collected, stored on ice, vortexed repeatedly, and then centrifuged at 12,000xg for 10 min at 4°C to remove cell debris. Protein concentration in each cell lysate supernatant was determined by the method of Bradford [47] or Brown et al [48]. Samples (25 μg cell lysate protein) were subjected to 10% SDS-PAGE [49] and Western analyses [50], as described [37] [45], by first detecting phospho-ERK1/2 (pERK1/2) and then stripping the transfer membranes to remove bound Ab and detecting total ERK1/2 (tERK1/2). To normalize protein load differences among wells, membranes were again stripped and reprobed with anti-actin Ab. Detection of bound primary Abs was with anti-goat or anti-rabbit IgG-HRP, as appropriate, and development using an enhanced chemiluminescence substrate and exposure to autoradiography film. Band densities were scanned into digital files and quantified as integrated densitometry values (*i*.*e*. the sum of all pixel values minus background correction) using an Alpha Innotech FluoroChem 8000 imaging system (Alpha Innotech Corp., San Leandro, CA). ERK1/2 activation is expressed as the ratio of pERK:tERK with the control treatment (with or without Hep) or time set as 100%. Each quantified signal for ERK1/2 activation was then normalized to the signal for Actin. For these signaling studies, replicate plates were incubated in the absence of ligand for up to 90 min, under the experimental conditions used, to verify that basal ERK1/2 activation levels were not significantly altered.

#### Co-Immunoprecipitation of ERK1/2 and HARE

Cells were grown to confluence, washed, collected and lysed as noted above. Goat Ab against V5, to detect HARE variants that contain the V5 epitope at the C-terminus (2 μg/ml), or nonimmune control goat Ab was added to cell lysate (300 μg protein) and incubated with rotation for 2 h at 4°C. Immune complexes were then collected by adding 30 μl of 250 μg/ml Protein A/G Plus Agarose resin and incubated overnight at 4°C with gentle mixing by rotation. The resin was washed three times with ice cold Lysis Buffer, the pellet was resuspended in 20 μl of 2x sample buffer [49], boiled for 3 min at 90°C and then subjected to SDS-PAGE, electro-transfer and Western blot analysis. Bound proteins were then detected by immunoblotting using anti-tERK1/2 Ab or anti-V5 Ab.

### Cell culture and LUC plasmid transfection

Transfection Medium contained DMEM with 8% FBS without antibiotics. Cells stably expressing HARE (WT or CD mutants) or EV were grown to near-confluence in Complete Medium, plated in 12-well tissue culture plates, and maintained in Complete Medium for at least 48 h prior to experiments. At 50–60% confluence, the medium was replaced with Transfection Medium 10 min prior to transfection. Transfection complexes were generated in serum-free medium by mixing Lipofectamine LTX and PLUS reagents with 1 μg/ml firefly LUC vector pGL4.32(luc2P/NF-κB-RE/Hygro) and 0.5 μg/ml *Renilla* luciferase vector (pRL-TK). Transiently transfected cells were grown for 18 h before use.

### Ligand stimulation of NF-κB activated gene expression

Cells were transiently transfected with firefly and *Renilla* LUC vectors as above, washed once each with sterile PBS and DMEM without serum, and then incubated in fresh serum-free DMEM for 1 h at 37°C. The medium was then removed and serum-free DMEM with the appropriate type and concentration of ligand was added, as indicated, and the cells were incubated at 37°C for 4 h. Time-zero values were in the absence of any ligand. The medium was removed by aspiration and cells were processed (below) to determine HARE-mediated NF-κB activated LUC gene expression. The cells were washed with sterile PBS, scraped and harvested in serum-free medium and centrifuged at 12,000 x *g* for 1 min. Supernatants were removed and cell pellets resuspended in 150 μl serum-free medium and assayed for LUC activity using a Dual-Luciferase Assay System following the manufacture’s protocol as described earlier [35]. The amount of firefly and *Renilla* LUC activity in each sample was measured and recorded as relative light units using a luminometer Glomax 20/20 (Promega). The ratio of firefly:*Renilla* LUC activities in each lysate sample was calculated and normalized to untreated cells as control (defined as 1.0). Results are expressed as the mean ± SEM fold-change in firefly:*Renilla* LUC activity.

### Statistical Analysis

Data are presented as the mean ± SEM based on three independent experiments, each performed in triplicate (n = 9). Western Blot data are presented as the mean ± SEM based on three independent experiments (n = 3). For statistical comparisons, data were first analyzed by a one-way ANOVA, and any significant difference in the group was then assessed by individual pair-wise post hoc Tukey’s HSD tests using GraphPad Prism v5 statistical software (GraphPad Software, Inc., San Diego). Pair-wise comparisons were made between EV and HARE cells treated with the same ligand concentration and then with HARE cells plus ligand *versus* EV cells without ligand. Only sample sets considered statistically significant in both cases are indicated with a symbol (*, p < 0.05; **, p < 0.005; ***, p < 0.001).

## Results

### HARE uptake of LMWH activates NF-κB mediated gene expression but only at high doses

**Fig 1:**
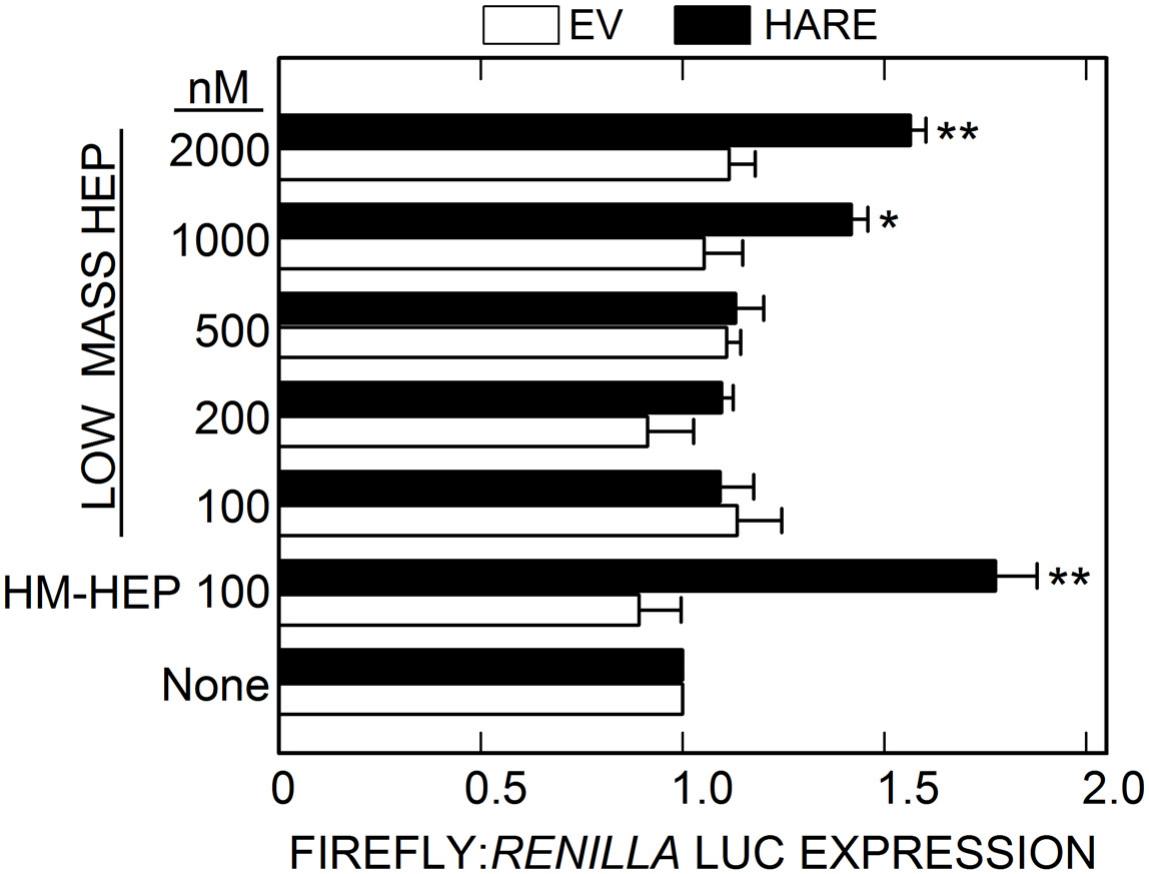
Low mass Hep stimulates HARE-mediated NF-κB activated gene expression less effectively than UFH. Cells expressing EV (white bars) or WT (black bars) HARE were grown and transfected with LUC recorder gene plasmids as described in Methods. The cells were incubated at 37°C for 4 h with 100 nM UFH or increasing concentrations of LMWH, as indicated, and then processed and analyzed for relative LUC activities. Values are the means ± SEM (n = 9) of the ratio of the two LUC activities determined from three independent experiments, with the value for unstimulated cells set as 1.0. Values for p compared WT cells with EV cells for each condition (*, p < 0.05; **, p 0.005) using ANOVA.

We previously found that 50 nM UFH stimulates ERK1/2 and NF-κB activation about ~2.5-fold compared to untreated WT or EV control cells [37]. The apparent K_m_ value (~20 nM) of this cellular response is consistent with the high binding affinity (K_d_ = 20–60 nM) of larger Hep for HARE [15]. Disrupting clathrin coated pit assembly greatly decreases signaling, indicating that HARE•Hep complexes are competent for signaling only after targeting to coated pits and endocytosis; cell surface complexes are unable to activate signaling. Since LMWH drugs are widely used to treat patients, it was important to determine if HARE binding to LMWH also stimulates ERK1/2 and NF-κB activation. Unlike UFH, HARE-mediated uptake of LMWH did not stimulate ERK1/2 phosphorylation (data not shown) in cells expressing WT HARE, even with 2.9 μM LMWH (10 μg/ml). This result was not surprising, based on the lower affinity of HARE for LMWH, and indicates that higher concentrations of LMWH might be required in order to detect ERK1/2 activation or that Western blot assays are not sensitive enough. In contrast, NF-κB activated gene expression was stimulated in WT, but not EV, cells incubated with increasing concentrations of LMWH (Fig 1). A significant dose-dependent increase in HARE-mediated NF-κB activation in WT cells occurred with a 1.5-fold stimulation at 2 μM LMWH (p < 0.005). Since LMWH is cleared by the renal system, whereas UFH is cleared by HARE-Stab2, *in vivo* these latter receptors would interact almost exclusively with UFH. The following experiments, therefore, utilized the more physiologically relevant UFH.

### ERK activation during HARE•Hep uptake requires the NPLY motif

**Fig 2:**
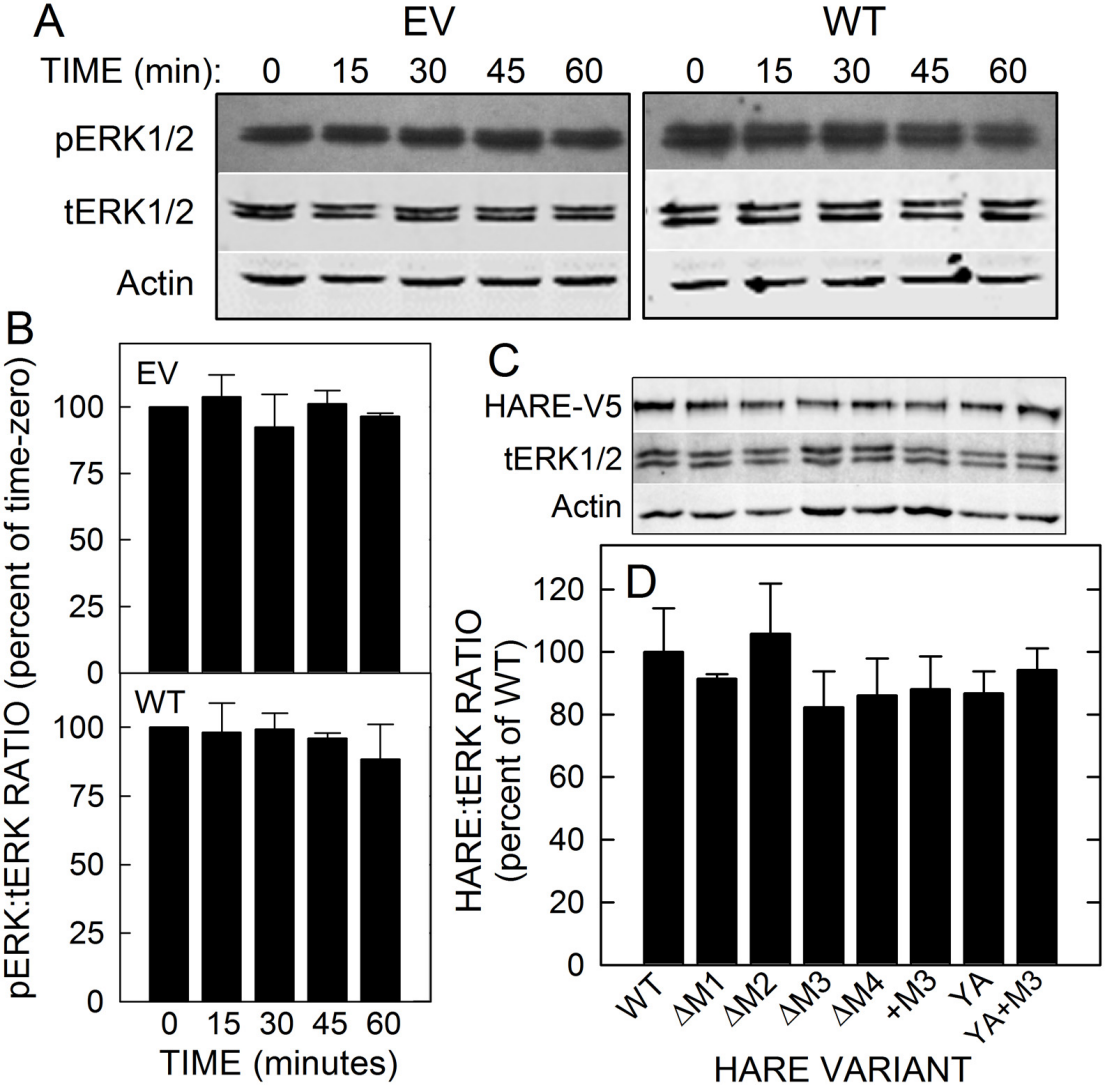
Basal ERK1/2 levels are stable and HARE expression levels are similar in all cell lines. A. WT and EV cells were cultured and then pre-incubated in serum-free medium for 1 h and further incubated for the indicated times in the absence of ligands. The cells were then processed to assess pERK1/2, tERK1/2 and actin as noted for the cell culture and Hep stimulation protocol in Methods. B. Blots (n = 3) were quantified and the pERK1/2:tERK1/2 ratios at each time were normalized to actin and are presented as percent ± SEM of the time-zero value. Normalized pERK/tERK ratios for either EV or WT cells were not significantly different from time-zero values at any time (p>0.05; unpaired Student’s t-test). C. Equal amounts of lysate protein from the indicated WT or mutant HARE cell lines were subjected to 8% SDS-PAGE, followed by Western blotting to detect HARE (with anti-V5 Ab), tERK1/2 and actin. D. Blots (n = 4) were quantified and the HARE and tERK levels, each normalized to actin content, are presented as a percent ± SEM of WT. No significant difference was found for the HARE or tERK content of any HARE variant compared to WT (p>0.05; unpaired Student’s t-test).

To determine which of the four HARE endocytic motifs (M1-M4) are important for downstream ERK1/2 activation, we used single-motif mutant cell lines in which one of these HARE CD motifs was deleted. Controls validating the experimental protocol used included showing that ERK activation levels in cells with EV (lacking HARE) or vector with WT HARE showed no significant changes (p>0.05) during 60 min of incubation in serum-free medium in the absence of Hep (Fig 2A and 2B). Thus, as expected ERK activation levels are at a constant background level (*i*.*e*. basal) in cells either not expressing HARE or expressing HARE but without a stimulating ligand. The experimental protocol and incubation conditions do not cause ERK activation. The levels of HARE, total ERK1/2 and actin were also assessed by Western blot analysis in the seven cell used (Fig 2C). Quantification of the results (n = 4), with each protein normalized to actin, showed that the ratios of HARE:tERK1/2 among the cell lines were not significantly different (p>0.05) than WT (Fig 2D).

**Fig 3:**
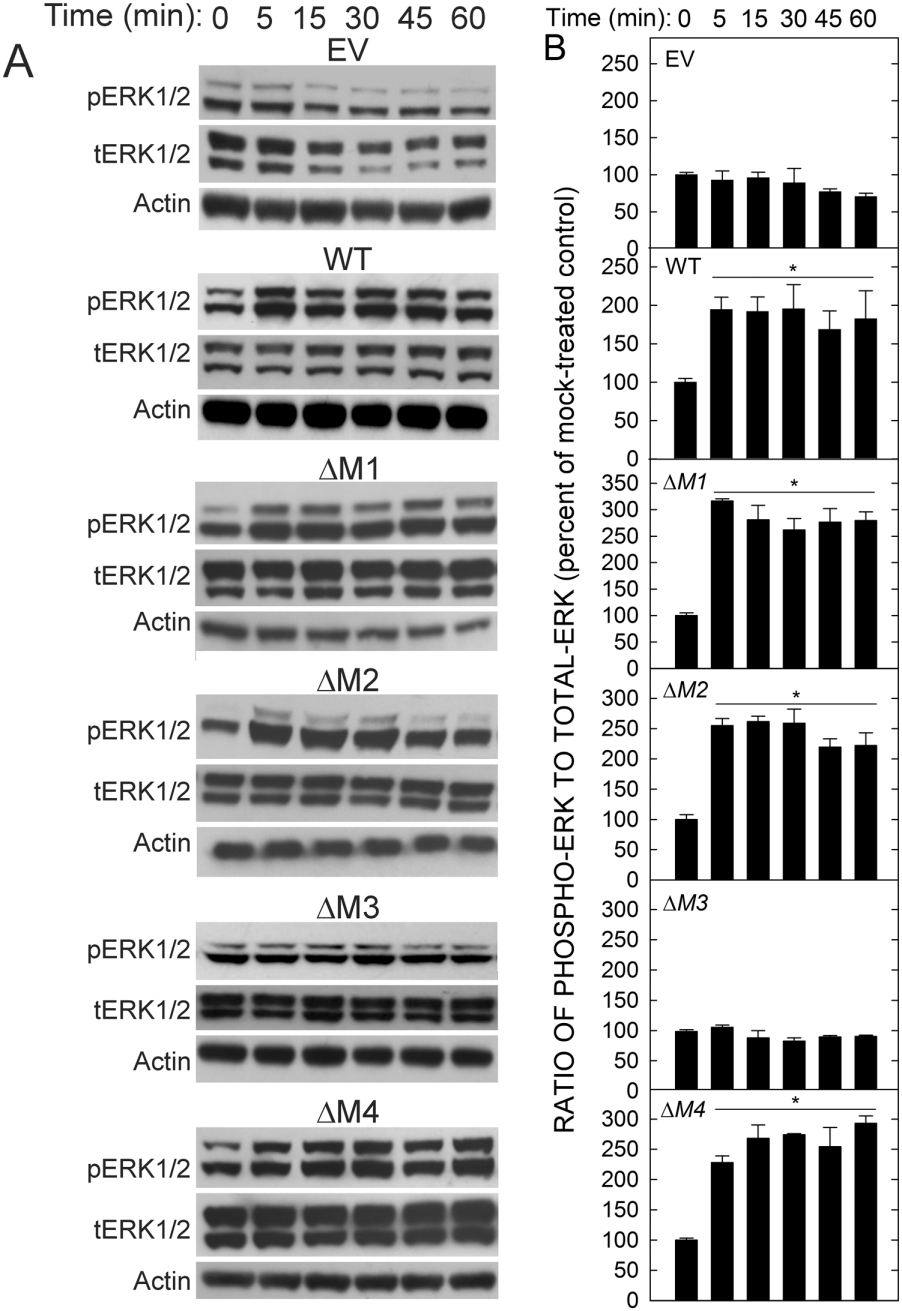
The *M3* motif is required for HARE•Hep-mediated ERK activation. Cells stably expressing EV or WT or the indicated HARE CD mutant (ΔM1, ΔM2, ΔM3 or ΔM4) were grown, processed and then incubated with 10 μg/ml (709 nM) UFH for 0–60 min, as indicated, and processed as described in Methods. A. Equal amounts of cell lysate (25 μg) were resolved in 10% SDS-PAGE and transferred to nitrocellulose membranes. Western blot analysis was performed with Ab against pERK1/2 (top panels). The same membranes were stripped and reprobed with Ab against tERK1/2 (middle panels) and then with anti-actin Ab (bottom panels). B. Blots from three independent experiments (n = 3) were digitized by scanning and densitometry analysis was performed to determine the pERK:tERK ratios, which were normalized to actin levels. The results are presented as mean percent ± SEM of the normalized pERK:tERK ratio compared with the time-zero (mock addition) value as 100%. An asterisk indicates that all time points for the WT, ΔM1, ΔM2, and ΔM4 samples (but not the EV or ΔM3 samples) were significantly different than their respective time-zero values (p < 0.05).

We recently found that three of these four motifs (M1, M3 and M4) are involved in coated pit mediated endocytosis of HARE•Hep complexes [42]. A different motif subset (M1, M2 and M3) are involved in uptake of HARE•HA complexes [41]. ERK1/2 activation in single-motif HARE mutant cells incubated with UFH was determined at different times by Western blot analyses to detect pERK1/2, tERK1/2 and actin (Fig 3A). HARE•HA mediated activation of ERK1/2 occurs in a bi-phasic manner with time, with an increasing response for up to 30 min and then a return to baseline by 45–60 min [36]. In contrast, quantification of replicate experiments (Fig 3B) showed that HARE•Hep activation of ERK1/2 reached maximal values by 5 min and remained at this level for at least an hour in WT cells and in cells expressing HARE ΔM1, ΔM2 or ΔM4 mutants. Deletion of M1, M2 (as expected) or M4 did not significantly alter ERK1/2 phosphorylation compared to WT; each cell line showed a similar time-course for a ~2–3 fold increase in ERK1/2 activation relative to time-zero, mock-treated control (p < 0.05 at each time). In contrast, deletion of the M3 motif abolished the ability of UFH to stimulate HARE-mediated ERK1/2 activation compared to WT or the three other single-motif deletant cell lines (Fig 3B). The results show that M3 is required for modulation of downstream signal transduction cascades initiated by HARE•Hep endocytosis.

### The M3 motif is required for HARE to form complexes with ERK1/2

**Fig 4:**
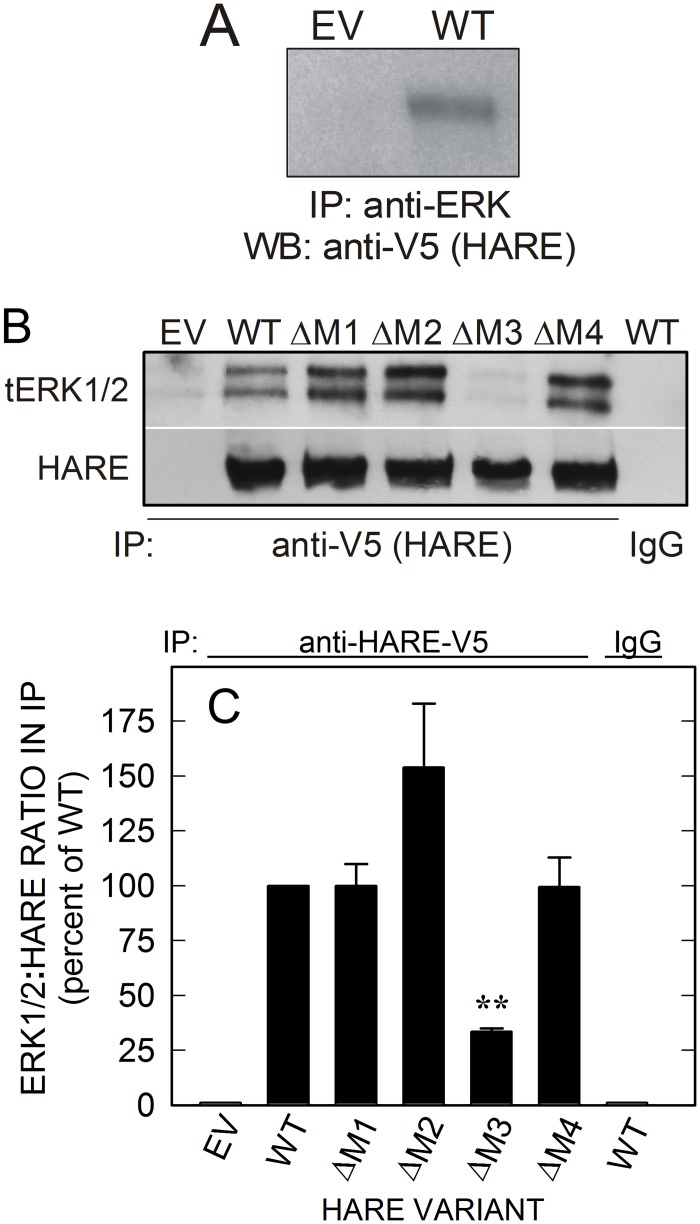
The HARE *M3* motif is required to form complexes with ERK. Cells stably expressing EV, WT or the indicated single-motif deletion HARE CD mutant were grown and processed as described in Methods and then incubated with or without 10 μg/ml (709 nM) UFH for 20 min and processed as in Fig 1. A. EV and WT cells were treated as in Fig 3 and Methods but in the absence of Hep or other ligands and cell lysates (300 μg of protein) were incubated with anti-tERK1/2 Ab. Immunoprecipitates were subjected to SDS-PAGE and Western blot analysis and blotted proteins were analyzed for HARE using anti-V5 Ab. B. HARE from equal amounts of lysate (300 μg of protein) from the indicated cells was incubated with goat anti-V5 Ab (2 μg/ml) and WT extract was also incubated with nonimmune control goat Ab (IgG). The immunoprecipitates were subjected to SDS-PAGE and Western blot analysis as described in Methods. Blotted proteins were analyzed using anti-tERK1/2 or anti-V5 Ab as indicated. C. Blots from independent experiments (n = 3), as in panel B, were scanned, digitized and densitometry analyses were performed to determine ERK:HARE ratios. The data are presented as mean ± SEM percent of the tERK:HARE ratio compared to WT as 100%. Samples with significant differences are indicated: **, p < 0.005.

We found previously that HARE and ERK1/2 form stable complexes in the absence, or the presence of HA, as detected by co-immunoprecipitation [36]. To determine if complexes between HARE and ERK1/2 are also present during HARE•Hep uptake, we treated WT or EV cells with or without a saturating amount of UFH (37-times apparent K_m_) and performed co-immunoprecipitation assays using Abs against HARE or tERK1/2 (Fig 4). HARE was detected using anti-V5 Ab to detect this C-terminal epitope. Western analysis of WT lysates showed that HARE co-precipitated with anti-ERK1/2 Ab in the absence of UFH (Fig 4A). Immunoprecipitates were prepared from WT and single-motif deletant lysates using anti-HARE-V5 Ab and blots were probed with anti-tERK1/2 Ab (Fig 4B). ERK1/2 was co-immunoprecipitated from cells expressing WT and HARE ΔM1, ΔM2, and ΔM4 CD mutants. ERK1/2 was not detected in immunoprecipitates from HARE(ΔM3) or EV cell lysates or in WT lysate immunoprecipitated with control nonimmune IgG. These results indicate that the observed complexes were specific and dependent on the presence of both HARE and ERK and that ERK1/2 also co-purified from cells expressing single-motif deletion mutants of HARE in which only M1, M2 or M4 were missing. Thus, HARE•ERK1/2 complex formation does not require these motifs (Fig 4C). Interestingly, ERK1/2 association with HARE(ΔM2) may be greater than WT, although this response was variable and not significant (p > 0.05). However, ERK1/2 association with HARE was decreased by 67% if the M3 motif was missing (p< 0.005). We conclude that M3 is the most important endocytic motif for HARE•ERK1/2 association and is required for normal cellular levels of complex formation. The decreased association of HARE(ΔM3) with ERK1/2 likely explains the inability to activate ERK1/2 (Fig 3B).

### The NPLY motif is required for HARE•Hep activation of NF-κB mediated gene expression

**Fig 5:**
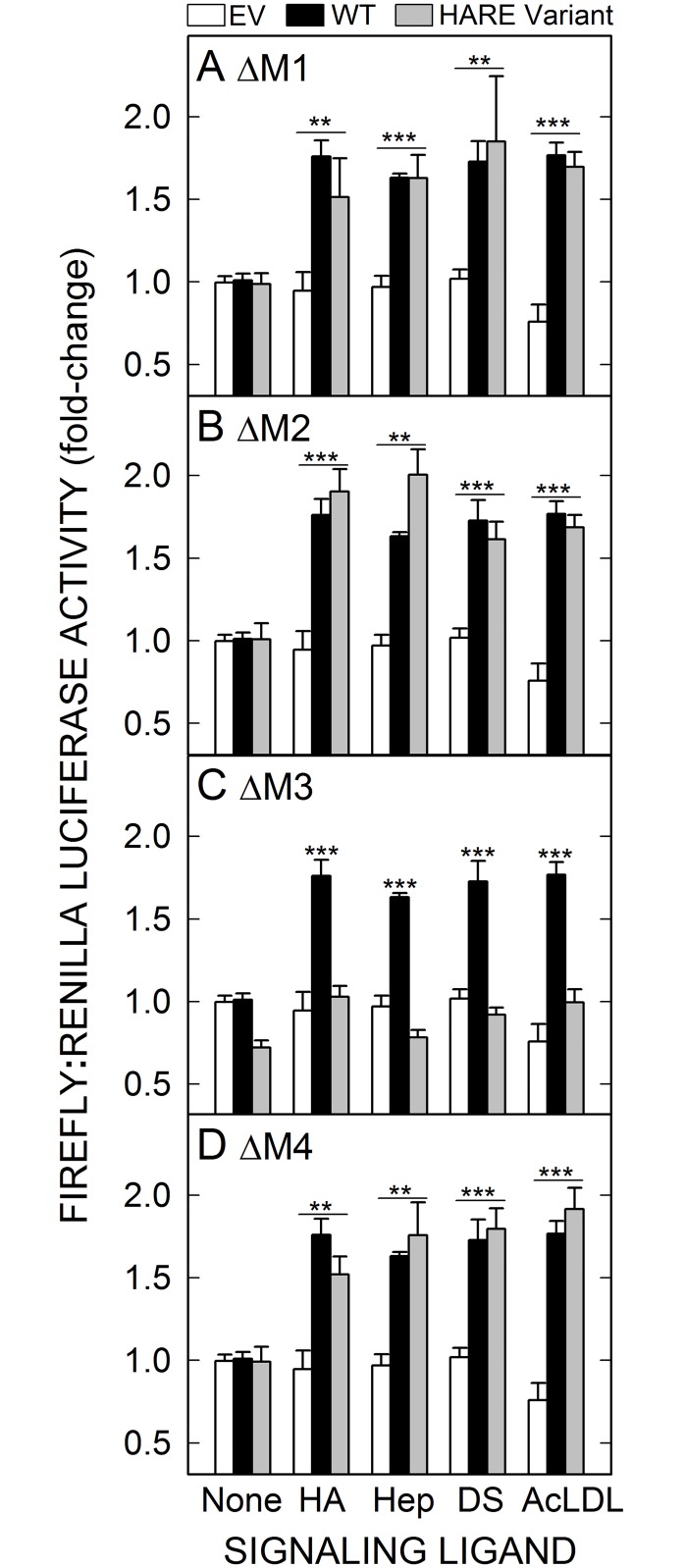
The HARE M3 motif is required for NF-κB activated gene expression by multiple signaling ligands. EV (white bars), WT (black bars) or cells expressing the indicated single-motif deletion HARE mutant (gray bars) were grown and transfected with LUC recorder gene plasmids as described in Methods. Cells were incubated at 37°C for 4 h with 100 nM HA, Hep, DS or AcLDL, as indicated, and then processed and analyzed for relative activities of the two LUC enzymes as in Fig 1. Values are means ± SEM (n = 9) from three independent experiments for HARE CD mutant cells: ΔM1 (A), ΔM2 (B), ΔM3 (C) or ΔM4 (D). Values for p are based on one-way ANOVA and pair-wise Tukey’s tests using pair-wise comparisons of HARE WT or mutant cells with EV cells for each ligand: **, p < 0.005; ***, p < 0.001.

We used single-motif deletion HARE-CD mutant cell lines to determine which, if any, of the four HARE endocytic motifs is required for cell signaling leading to NF-κB activation. Dual-luciferase reporter assays were performed with four different signaling ligands [37]; HA, Hep, DS and AcLDL. Deletion of M1, M2 or M4 did not inhibit NF-κB activation and gene expression in the presence of any of these ligands (Fig 5A, 5B and 5D). Activation mediated by these single-motif deletion mutants was similar to the activation in WT cells. As expected, LUC gene expression in response to each of the four signaling ligands was significantly higher in WT or these three mutant cells than in EV cells (p ≤ 0.005). In contrast, none of the four signaling ligands stimulated NF-κB activation or gene expression in HARE(ΔM3) cells (Fig 5C). These results are consistent with the results seen for ERK1/2 activation (Fig 4B), indicating that the M3 motif is also required for activation of HARE-mediated downstream cell signaling pathways leading to NF-κB activation.

### Tyr^2519^ in Motif 3 is required for HARE•Hep mediated activation of ERK1/2

Many studies have identified Tyr as the most important amino acid for NPXY function, since it is usually phosphorylated in order to interact with cytoplasmic proteins required for signaling that contain SH2 domains; these domains bind phospho-tyrosine (pTyr) within NPXY motifs [51–53]. To verify that this amino acid in M3 of the HARE CD is needed for cell signaling, we generated two stable HARE-expressing cell lines. One mutant contained a substitution of Tyr^2519^ to Ala with no other CD changes; a HARE(Y2519A) mutant. The same Y2519A change was also made in the M3-only CD mutant (*i*.*e*. ΔM1M2M4), in which only M3 is present; HARE[M3-only(Y2519A)]. Previous endocytosis assays with HA [41] and Hep [42] revealed that the Y2519A change alone, in a WT HARE background, does not impair endocytosis, whereas cells expressing HARE[M3-only(Y2519A)] show completely impaired endocytosis. Thus, in the absence of other endocytic motifs, Tyr^2519^ in M3 is required in order to mediate HARE•ligand targeting to coated pits.

**Fig 6:**
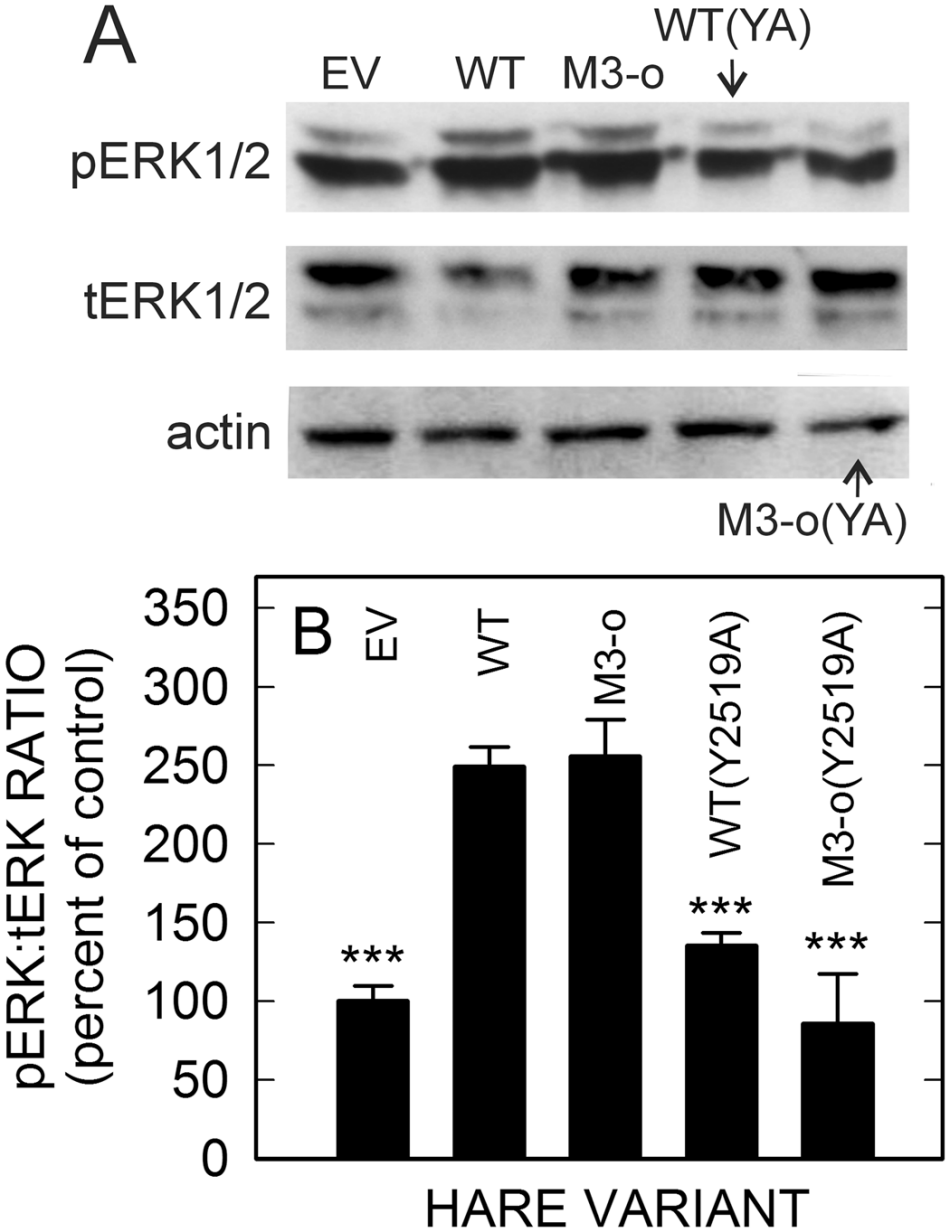
Mutation of Tyr^2519^ in M3 decreases or eliminates HARE•Hep-mediated ERK1/2 activation. Cells stably expressing EV, WT, or the M3-only (M3-o in panels), M3-only(Y2519A) or WT(Y2519A) HARE mutants were grown and processed as described in Methods and then incubated with 709 nM UFH for 20 min and further processed as in Fig 3. A. Western blot analyses show pERK1/2 (top panel), tERK1/2 (middle panel) and actin (bottom panel). B. Blots from independent experiments (n = 3) were scanned, digitized and densitometry analyses were performed to determine pERK:tERK ratios. The data, normalized to actin content, are presented as mean percent ± SEM of the pERK:tERK ratio compared with EV cells as 100%. Samples with significant differences to WT are indicated: ***, p < 0.001.

To assess the role of Tyr^2519^ in HARE•Hep-initiated ERK1/2 signaling, we incubated EV, WT, M3-only, and the two Tyr-to-Ala mutant cells with UFH, and then determined pERK1/2 and tERK1/2 levels by Western analysis (Fig 6A). Quantification of replicate blots (Fig 6B) showed 2.5-fold stimulations of ERK1/2 activation in WT and M3-only cells compared to EV cells (p<0.001), whereas UFH-mediated stimulation was eliminated in M3-only(Y2519A) cells and was decreased by 75% in WT(Y2519A) cells.

### Tyr^2519^ in the M3 motif is required for HARE mediated activation of NF-κB mediated gene expression in response to four signaling ligands

**Fig 7:**
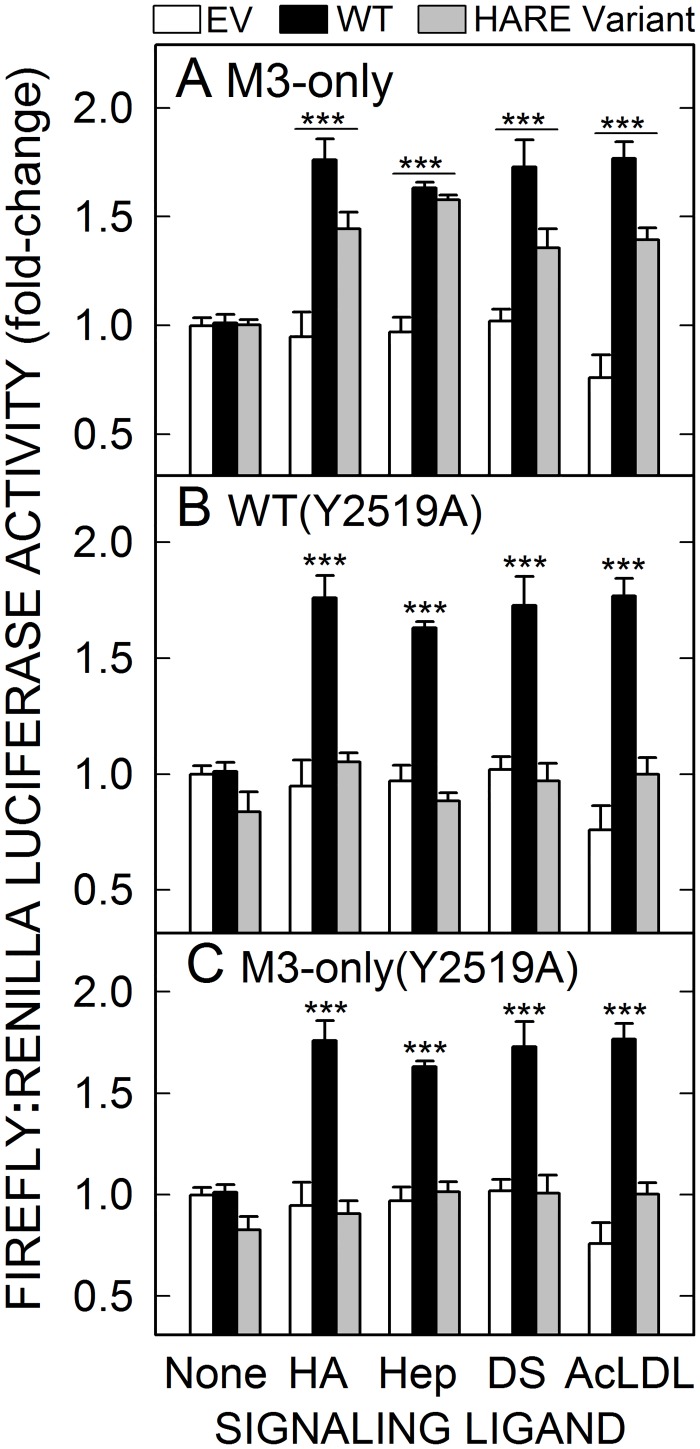
Tyr^2519^ in HARE M3 modulates NF-κB activation and gene expression in response to endocytosis of multiple ligands. Cells expressing EV (white bars), WT (black bars) or the indicated mutant (gray bars) HARE were grown, transfected, and incubated at 37°C for 4 h as in Fig 5 with 100 nM of one of the four signaling ligands: HA, Hep, DS or AcLDL. All cells were processed, and analyzed for relative LUC activity as in Fig 1: M3-only, (A); WT(Y2519A), (B); or M3-only(Y2519A), (C). Values are means ± SEM (n = 9) of the indicated ratio of the two LUC enzymes from three independent experiments, with the value of mock-treated cells as 1.0. Values for p are based on one-way ANOVA and pair-wise Tukey’s tests using pairwise comparisons of HARE WT or mutant cells with EV cells for each ligand: ***, p < 0.001.

To assess the general role of Tyr^2519^ in NF-κB activation, the five cell lines used in Fig 6 were incubated with Hep, HA, DS or AcLDL and reporter LUC gene expression was measured (Fig 7). Cells expressing the M3-only CD mutant showed significant activation of gene expression (p<0.001) with each of the four signaling ligands compared to EV cells or WT cells not exposed to ligand (Fig 7A). NF-κB activation of gene expression was slightly decreased, although not significantly, in M3-only cells exposed to HA, DS or AcLDL, but activation was significantly increased in each case compared to EV (p < 0.005). In contrast, increased NF-κB mediated gene expression did not occur in the presence of any of the four signaling ligands in either WT(Y2519A) cells (Fig 7B) or M3-only(Y2519A) cells (Fig 7C). Thus, the two CD Tyr^2519^-mutants are not competent to initiate HARE•ligand mediated NF-κB activation.

## Discussion

NPXY motifs, which are phosphorylated when functional, are present in the CDs of many cell surface receptors (*e*.*g*. those for EGF, insulin, and HARE/Stab2) and interact with the pTyr-binding SH2 domains of cytosolic signaling proteins [53]. NPXY motifs also target receptor•ligand complexes to coated pits for rapid internalization [41,54,55]. These early interactions within signaling cascades ultimately lead to the control of multiple cellular responses such as cytoskeleton organization, cell survival, proliferation and differentiation [56,57]. Mutation of these NPXY sequences results in decreased internalization of receptor•ligand complexes [41,54]. For example, CS-1 cells expressing NPXY sequence mutants of integrin α5β3 lose their biological functions including attachment, spreading and migration on vitronectin-coated plates [58].

The results reported here demonstrate that of the four HARE CD endocytic motifs involved in coated pit targeting of UFH and other ligands, only the NPXY motif (M3; NPLY) is required for the ability to activate ERK1/2 and NF-κB signaling pathways (Figs 3 and 5). The NPLY motif was also critical to form stable HARE•ERK1/2 complexes (Fig 4), which occurs in the absence of ligand [36], and are thus primed to be rapidly deployed in downstream cell signaling. We found that Tyr^2519^ in the M3 motif is needed for both the activation of ERK1/2 (Fig 6) and NF-κB mediated gene expression (Fig 7). These results indicate that Tyr^2519^ in M3 can function as a docking site for other cytosolic proteins to activate downstream cell signaling pathways. The pTyr group in M3 is needed for ERK and NF-κB activation stimulated by ligand binding and uptake, as well as any HARE-dependent contribution to basal activation of these signaling cascades. The four HARE CD endocytic motifs are redundant in terms of supporting efficient coated pit targeting for multiple ligands. Thus, while only the M3 motif mediates both Hep uptake and intracellular signal transduction, the M1 and M2 motifs also mediate the Hep clearance function.

The mechanism for clearance of subcutaneously injected Hep from the body depends on its mass and binding affinity for plasma proteins [59] and HARE, which is the systemic clearance receptor for Hep [15,16]. Recombinant cell lines expressing HARE, primary rat liver SECs or purified recombinant HARE [15] bind to larger UFH (13–17 kDa) with high-affinity (K_d_ ~20 nM) and bind to smaller LMWH (3.5 kDa) with much lower affinity (K_d_ ~10 μM); HARE binding affinity for larger Hep is 500-to-1,000 fold greater than for smaller Hep. Since smaller Hep is effectively cleared by a renal mechanism, its lower affinity for the main Hep clearance receptor does not present a problem physiologically for it to be cleared and removed rapidly.

In addition to the primary clearance function of HARE and Stab2 to remove and degrade multiple ligands, sometimes simultaneously [17], an unexpected finding was that ligand uptake can also stimulate cell signaling pathways that activate ERK1/2 and NF-κB. Phagocytosis of apoptotic cells by activated macrophages is mediated by HARE/Stab2 binding to phosphatidylserine and this interaction stimulates signaling leading to the synthesis and release of TGF-β, an anti-inflammatory cytokine [28]. We found that HARE-mediated HA uptake stimulates activation of ERK1/2 [36] and also NF-κB [35], which leads to gene expression changes. HARE•HA-mediated NF-κB activation is strikingly dependent on HA mass, occurring only with HA of 40–400 kDa, despite the fact that all sizes of HA >2 kDa are endocytosed. Internalization of HARE•Hep complexes also stimulates activation of ERK1/2 [45] and NF-κB [37]. Another surprising finding was that only a subset of ligands is capable of stimulating ERK1/2 and NF-κB signaling [37]. In addition to phosphatidylserine noted above, HA, Hep, DS and AcLDL are all able to activate both signaling pathways. In contrast, chondroitin sulfates type A, C, D and E do not stimulate either ERK1/2 or NF-κB signaling. Furthermore, HARE•HA signaling requires the presence of a complex N-glycan at Asn^2280^in order to occur [46]. Cells expressing a HARE(N2280A) mutant fail to signal in response to ongoing HA uptake, whereas Hep, DS and AcLDL all activate ERK1/2 and NF-κB pathways normally. Thus, HA signaling proceeds by a different mechanism than signaling mediated by other ligands. Although as yet known, there is presumably biological significance to both the very narrow HA size-dependence for signaling and the competence of only some ligands to activate signaling.

These above observations indicate that additional consequences are involved upon binding some, but not other, ligands. For example, ligand binding could stimulate protein oligomer formation altering HARE conformation to induce interaction with another protein, and the new complex could then activate the already bound ERK. Another possible mechanism is that conformational changes in the external HARE domain due to ligand binding are communicated via the transmembrane domain to alter conformation of the cytoplasmic domain and to activate bound ERK.

Without ligand present, HARE is already in complexes with ERK, JNK and p38 [36]. Many receptors are normally in preformed complexes with signaling proteins, in order to create more rapid and controlled responses to the stimuli monitored by that receptor. The existence of such complexes is strong evidence for a potential signaling role for the receptor. Especially for constitutively recycling clearance receptors, such as HARE, that constantly recycle every 7–10 min [31,45] along a spatial and temporal pathway among multiple cellular compartments (*e*.*g*. plasma membrane, early and late endosomal compartments and CURL), it makes sense that signal-competent complexes are already assembled and ready to respond when bound ligand-receptor complexes are internalized via a coated pit pathway. Many currently understood signaling cascades consist of preformed complexes that are activated intracellularly during internalization and endosomal trafficking [60,61]. Importantly, unlike hormone receptors, systemic clearance receptors are constantly exposed to their ligands and their signaling cascades are likely designed to sense changes in ligand levels, rather than just the steady-state presence of the ligand [29]

A significant question that remains unanswered is whether the HARE-mediated NF-κB and ERK1/2 activation pathways are linked. Attempts to answer this important question by using specific agents such as MEK inhibitors failed because both ligand-induced and TNF-α-induced NF-κB activations were blocked by the necessary solvents, DMSO or ethanol; see supplemental figure S4AB in reference [35]. Others have also reported that DMSO inhibits HA-fragment induced NF-κB activation of inflammatory gene expression in mouse alveolar macrophage and epithelial cells [62].

The redundancy of multiple endotcytic motifs in the HARE CD for targeting Hep to the coated pit pathway indicates that this HARE clearance function is of primary importance. Similarly, HA uptake by single-motif deletant mutants showed that a different subset of three motifs (M1, M3, and M4) is also involved in total HA uptake [41]. Thus, for both HA and Hep, each competent motif contributes to the total ability of HARE to mediate ligand uptake. The loss of one motif, or even two, decreases but does not eliminate the ability of HARE to mediate uptake of HA or Hep. In contrast, only the shared M3 motif is necessary and sufficient for both ERK1/2 and NF-κB signaling in response to HA or Hep endocytosis. Although it has not been directly tested, it is likely that a similar motif redundancy as seen for HA and Hep uptake also exists for the HARE-mediated uptake of the two other ligands examined here; DS and AcLDL. That the signaling functions of HARE•ligand uptake are only associated with the NPLY motif and that not all endocytosed ligands stimulate signaling also indicate that clearance is the primary function of HARE.

Although signaling may be an evolutionally later or secondary HARE function, it is likely to be important nonetheless. We previously proposed that HARE signaling in response to multiple ligands constitutes a systemic Tissue-Stress Sensing System designed to monitor the turnover of components arising from different tissues, including dead cell debris and matrix structural molecules [29]. In response to changes from the normal homeostatic levels of these recorder molecules (*e*.*g*. due to injury or infection), lymph node and liver SECs and circulating macrophages would secrete newly made cytokines and other factors to help affected tissues battle the stressing factor(s). If this proposed feedback system can be confirmed and understood, then there could be opportunities for development of strategies to enhance or diminish the normal responses. Further studies are needed to determine what other HARE ligands are also internalized using multiple motifs and are competent to stimulate signaling.
